# Intraventricular WHO Grade 3 Pleomorphic Xanthoastrocytoma: A Rare Case Report and Review of the Literature

**DOI:** 10.1155/crip/9992805

**Published:** 2025-04-28

**Authors:** Mohamed Alhantoobi, Nadeen Alkhoori, Euan Zhang, John Provias, Kesava Reddy

**Affiliations:** ^1^Department of Neurosurgery, Hamilton General Hospital, McMaster University Medical Centre, Hamilton, Ontario, Canada; ^2^Department of Neurosurgery, Zayed Military Hospital, Abu Dhabi, UAE; ^3^Department of Radiology, Hamilton General Hospital, McMaster University Medical Centre, Hamilton, Ontario, Canada; ^4^Department of Pathology & Molecular Medicine, Hamilton General Hospital, McMaster University Medical Centre, Hamilton, Ontario, Canada

**Keywords:** case report, intraventricular tumor, Neurofibromatosis Type 1, pleomorphic xanthoastrocytoma, WHO Grade 3 pleomorphic xanthoastrocytoma

## Abstract

**Background:** Cerebral pleomorphic xanthoastrocytoma (PXA) in patients with Neurofibromatosis Type 1 (NF1) is truly a rare entity. Intraventricular anaplastic PXA (APXA) is an even more uncommon presentation, with only three cases reported in the literature.

**Case Description:** We present the case of a 30-year-old female with known NF1 who developed an intraventricular WHO Grade 3 PXA. The tumor was initially resected but recurred aggressively, requiring further surgery and adjuvant therapy with radiation, lomustine, and bevacizumab. Despite treatment, the tumor continued to progress, and the patient's clinical course deteriorated.

**Discussion:** Distinguishing Grade 3 PXA from epithelioid glioblastoma can be diagnostically challenging and often requires further molecular testing. Aggressive multimodal therapy including maximal safe resection, radiation, and chemotherapy may be warranted, but outcomes remain poor. The challenging location of this patient's tumor in the ventricular system added to the complexity of overall treatment. Furthermore, the association of WHO Grade 3 PXA with NF1 is exceedingly rare, and the optimal management and prognosis of this rare tumor in the setting of NF1 are not well established.

**Conclusions:** This case report highlights the unique challenges in diagnosing and managing intraventricular WHO Grade 3 PXA, particularly in the context of NF1. Additional research is necessary to enhance the understanding and effective management of these rare and aggressive tumors.

## 1. Introduction

Cerebral pleomorphic xanthoastrocytoma (PXA) in patients with Neurofibromatosis Type 1 (NF1) is an exceedingly rare entity. Generally, PXA is a benign lesion; however, its prognosis in association with NF1 is not yet well established. In the literature, there are a few reported cases of cerebral PXA, mostly in the supratentorial compartment [1]. Other locations include the cerebellum, cerebellopontine angle, pineal region, and the ventricular system [1].

Based on the available literature, there have been only three cases of intraventricular WHO Grade 3 PXA reported. Furthermore, Grade 3 PXA exhibits a more aggressive behavior and requires differentiation from other forms of high-grade gliomas, such as IDH wild-type glioblastomas [1, 2]. The histopathological characteristics of PXA WHO Grade 3 such as epithelioid features closely resemble those of epithelioid glioblastoma (EGB), and distinguishing between the two can pose a diagnostic challenge that often requires genetic testing [3]. This differentiation is critical because Grade 3 PXA is associated with significantly greater survival benefits from targeted therapies, such as MAPK pathway inhibitors [4].

In this case report, we describe a case of intraventricular anaplastic PXA and review the available literature.

## 2. Case Presentation

**Figure 1:**
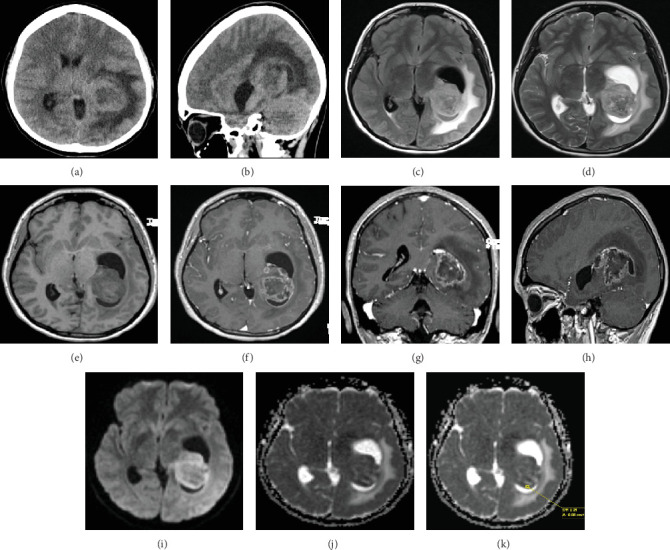
Left intraventricular heterogeneous lesion with temporal horn entrapment. (a, b) A 5-cm heterogeneous intra-axial mass centered in the region of the trigone of the left lateral ventricle. Moderate extent of vasogenic edema. Entrapment of the temporal horn of the left lateral ventricle. There is significant mass effect causing a midline shift of 9 mm and diffuse sulcal effacement. (c, d) FLAIR and T2 MRI images confirm the presence of intra-axial mass exhibiting heterogeneous signal on T2-weighted images causing entrapment of the temporal and occipital horns of the left lateral ventricle. There is a moderate extent of vasogenic edema within the surrounding left temporoparietal region. (e, f) 3D T1-weighted images pre- and postgadolinium. Pre-gad, the tumor exhibits hypointense to isointense signal. Post-gad, the tumor exhibits heterogeneous enhancement. There is thin ependymal enhancement of the surrounding trigone of the left lateral ventricle. (g, h) In coronal and sagittal postgadolinium images, the tumor appears to be arising from the left temporo-occipital region extending into the trigone of the left lateral ventricle. The left choroid plexus is displaced superiorly. There is nodular extension of nonenhancing tumor anteriorly into the left hippocampus, which exhibits volumetric expansion. (i–k) DWI and ADC MRI sequences reveal a heterogeneous diffusion restriction with ADC values reaching as low as 511, indicative of high grade.

A 30-year-old female known case of NF1 (diagnosed at 2 years old) and history of stable right optic nerve glioma (diagnosed at 9 years of age) followed radiographically presented with a 3-week history of left-sided headache and nausea with vomiting. The patient initially attributed her symptoms and headache to her usual migraines; however, upon failure of the migraine headaches to subside, she presented to the emergency department. Initial investigations with head CT scan followed by subsequent brain MRI with gadolinium demonstrated a large infiltrative tumor centered in the trigone of the left lateral ventricle with enhancing and nonenhancing components (Figure 1). The patient's symptoms improved dramatically with initiation of dexamethasone therapy. She was then scheduled urgently for left transcortical parieto-occipital craniotomy to resect the tumor. Postoperative course was complicated by postoperative delirium and SIADH which resolved by reducing the dexamethasone dose and infusion of hypertonic saline, respectively.

Postoperative MRI images were consistent with small focus of residual tumor, particularly along the hippocampal region with some encroachment on the insula. Complete neural axis MRI did not reveal evidence of disseminated disease. Of note, the patient did not have any previous radiation therapy or any other adjuvant treatment for her right optic glioma.

**Figure 2:**
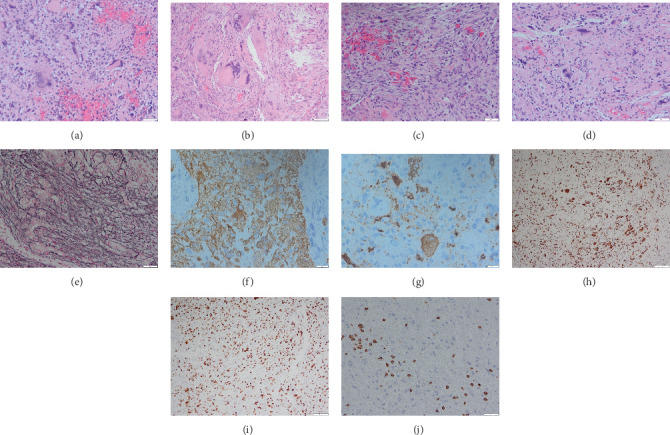
Intraventricular region tumor pathology. Representative tumor histology H/E sections (a–d) showing pleomorphic large tumor cells, with some xanthomatous change (b). (e) Heavy reticulin deposition in the tumor. Immunohistochemistry of the tumor; GFAP positive (f), with some neuronal differentiation, synaptophysin-positive rare cells (g). ATRX is retained indicating wild-type state (h), very high proliferation shown with Ki-67 (i). Areas of reactive T lymphocytes CD 3 positive (j).

Pathology of the surgical resection specimens showed a highly pleomorphic glioma with bizarre larger cells, nuclear inclusions, multinucleated cells, and some xanthomatous change (Figure 2). Focal areas had a more spindled appearance. Synaptophysin suggested focal neuronal differentiation as is frequently seen in PXA. High-grade features were increased mitotic count up to 8 per 10 high-power fields and tumor necrosis. The tumor was fully isocitrate dehydrogenase wild type, strongly glial fibrillary acidic protein positive with extensive reticulin deposition and an inflammatory T cell infiltrate. Proliferation was high as shown by Ki-67 (20%–40%). Alpha-thalassemia/mental retardation (ATRX) showed retained nuclear immunopositivity which also indicates wild-type state. P53 showed nuclear positivity in scattered lesional cells but overall was interpreted as negative. P16 stained several small foci of tumor cells but was largely negative. In summary, this confirmed the diagnosis of IDH-negative, MGMT unmethylated PXA WHO Grade III.

Further molecular testing shows oncogenic single nucleotide variations (point mutations) in P53 and NF1, as well as RB1. No fusion gene transcripts were detected. A number of additional pertinent negatives are also shown including the lack of any mutation involving ATRX, IDH1, IDH2, P1K3R1, EGFR, BRAF, p10, histo and protein 1H3B, H3, H3F3A, PDGFRA, and CCDN2.

The patient recovered well postoperatively with a Karnofsky score (KPS) of 80. The case was discussed in the neuro-oncology tumor board where the consensus was to proceed with adjuvant radiotherapy delivering 5940 cGy in 33 sessions.

Five weeks after surgery, the patient presented to the emergency department with headache associated with nausea and vomiting and was noted to have Gerstmann's syndrome, right homonymous hemianopia, and altered mental status on examination. Urgent imaging showed significant increase in residual tumor size with acute hydrocephalus secondary to entrapment of left temporal horn. In view of the dramatic clinical changes, the patient was taken for urgent reopening of the left parieto-occipital craniotomy and redo resection of recurrence using the METRx tubular retractor system modified for cranial use [5]. Subtotal resection was achieved as evident on postoperative MRI scan. The patient recovered well and discharged a few days later. Of note, the patient had incomplete resolution of Gerstmann's syndrome and right homonymous hemianopia. Adjuvant therapy in the form of lomustine and bevacizumab was started after her discharge.

Furthermore, the patient had further disease progression and therefore was approved for MEK inhibitor, trametinib. However, after 2 weeks of initiating trametinib treatment, it was stopped due to significant increase in liver enzymes and subsequently started on bevacizumab as a palliative measure as her clinical course continued to deteriorate.

## 3. Discussion

This submission is intended to present a rare disease entity and review the literature on intraventricular WHO Grade 3 PXA. In general, intraventricular tumors are uncommon, representing only 0.8%–1.6% of all intracranial tumors [6]. These tumors are predominantly benign and can be categorized into primary and secondary types [6]. Primary intraventricular tumors originate from the ependymal and subependymal lining, choroid plexus, and septum pellucidum [6]. The choroid plexus, due to its rich blood supply, is the most frequent site for metastatic lesions in this region. Secondary tumors occur when adjacent brain tumors invade the ventricular area [6].

PXAs are rare, low-grade (WHO Grade II) astrocytic tumors that typically occur in the cerebral hemispheres of young adults. However, PXAs have also been reported in other locations such as the hypothalamus, spinal cord, sella, cerebellum, and retina, though these are uncommon [7, 8]. PXAs are generally associated with a good prognosis after complete surgical resection [9].

WHO Grade III PXA is a more aggressive subtype characterized by increased mitoses, vascular proliferation, and/or necrosis [3]. They can be difficult to distinguish from other high-grade gliomas like anaplastic astrocytomas and glioblastomas, especially the epithelioid variant (EGB); however, histology appearance as methylation profiling helps in distinguishing both entities to a large degree [3, 10].

Only a few cases of intraventricular PXAs have been reported, and to the authors' knowledge, this case represents only the fourth documented report of a Grade 3 PXA occurring within the ventricular system [4, 6, 11]. The intraventricular location may have contributed to the early recurrence of the tumor in this case, possibly due to microscopic residual disease involving the ependymal lining as well as to the technical challenges of obtaining gross total resection due to its deep location [11].

The optimal management of intraventricular Grade 3 PXAs is not well established due to the rarity of these tumors [8]. This patient underwent adjuvant radiation and chemotherapy in order to achieve longer-term control as proposed in some previous cases [12, 13].

The fifth edition of the *WHO Classification of Tumors of the Central Nervous System* acknowledges the significant morphological and genetic similarities between EGB and PXA, making their distinction challenging, particularly without a prior history of a WHO Grade 2 PXA [3]. However, certain histological features can help differentiate between these entities. Notably, the presence of a dense reticulin network is more characteristic of PXA and is not typically observed in EGB. When considering the overall constellation of findings, including histological, immunohistochemical, and molecular features, a diagnosis of WHO Grade 3 PXA may be favored over EGB in some cases [3]. It is important to note that the current WHO classification recognizes the diagnostic complexity in distinguishing between these tumor types, especially in cases where there is no previous history of a lower-grade PXA [3]. This underscores the need for a comprehensive evaluation of all available clinical, radiological, histological, and molecular data to arrive at the most accurate diagnosis.

Central nervous system (CNS) WHO Grade 2 PXA generally has a favorable prognosis, with over 70% of patients surviving for 10 years or more [4, 11]. However, these tumors can recur and progress, emphasizing the critical importance of early and complete surgical resection [4, 11]. Malignant transformation to WHO Grade 3 PXA occurs in approximately 10%–20% of cases [4, 11]. This progression can happen within a wide timeframe, ranging from as short as 7 months to as long as 15 years after the initial diagnosis [4]. For patients diagnosed with CNS WHO Grade 3 PXA, treatment typically involves a multimodal approach [4]. Following gross total resection, adjuvant therapy is recommended, which may include both radiotherapy and chemotherapy [4]. Additionally, close surveillance is crucial for monitoring potential recurrence or further progression. WHO Grade 3 PXA can develop through two pathways: either as a progression from a lower-grade PXA (WHO Grade 2) or as a de novo presentation. The prognosis for WHO Grade 3 PXA is generally poor, with studies reporting a 5-year survival rate of 57.1% [8].

BRAF V600E mutations, which lead to the constitutive activation of the RAS/RAF/MEK/ERK signaling pathway, are present in 38%–60% of patients with PXA [14]. Specifically, these mutations are found in 17%–65% of Grade 3 PXAs and 50% of EGBs [14]. Despite their prevalence, BRAF V600E mutations are not a diagnostic criterion for PXA lesions [3]. The introduction of BRAF inhibitor therapies, such as dabrafenib and trametinib, for patients with BRAF V600E mutant gliomas has demonstrated favorable clinical and radiographic responses, showing durable antitumor activity [15]. Vemurafenib monotherapy showed an objective response rate of 42.9% for patients with BRAF V600E mutant PXA, compared to 9.1% for those with glioblastoma in the IDH wild-type subgroup [16, 17]. Overall, case reports indicate that treatment regimens combining BRAF and MEK inhibitors can result in up to 35 months of stable disease for patients with BRAF V600E-mutated PXA [16, 17].

Individuals with the genetic disorder NF1 have an increased risk of developing various types of brain tumors, particularly low-grade gliomas [18]. One of the most common brain tumors in NF1 patients is optic pathway gliomas, which are slow-growing pilocytic astrocytomas that can involve the optic nerves, chiasm, or tracts, often leading to vision problems [3, 18]. Brainstem gliomas, typically located in the medulla or cervicomedullary region, are another frequent occurrence, exhibiting a more indolent course compared to their non-NF1 counterparts [19]. Low-grade gliomas can also arise in the cerebral hemispheres, cerebellum, thalamus, or spinal cord, causing various neurological deficits [19]. While these low-grade tumors predominate in children with NF1, adults with this condition have a higher risk of developing high-grade gliomas, such as glioblastomas, which are aggressive and more malignant tumors affecting the cerebral hemispheres [18, 19].

In this paper, we reviewed the literature and identified three previously reported cases of intraventricular Grade 3 PXA. Those findings are summarized in Table 1. We used the following terms to search PubMed: anaplastic, high grade, pilocytic astrocytoma, intraventricular, and ventricular. Initial result identified 90 cases; however, three of them were relevant as summarized in Table 1.

In conclusion, this case highlights the unique diagnostic and management challenges posed by intraventricular WHO Grade 3 PXA, particularly in the context of its rarity and aggressive behavior. The intraventricular location may contribute to early recurrence, and the overlap with EGB can be diagnostically challenging. While complete surgical resection is the mainstay of treatment, adjuvant therapies including radiation, chemotherapy, and targeted agents like BRAF inhibitors may be considered for Grade 3 lesions. In addition, the patient's underlying genetic disorder of NF1 presented an additional challenge in the overall management. Overall, the patient outcome remains poor, and further research is needed to better understand the optimal treatment approaches and prognosis for this rare tumor subtype.

## 4. Conclusion

This case report highlighted a rare and challenging disease entity in terms of initial diagnosis and subsequent surgical and oncological management. The complexity of differentiating between similar tumor types, such as PXA and EGB, underscores the necessity for comprehensive diagnostic approaches that integrate histological, immunohistochemical, and molecular data. The presence of BRAF V600E mutations and the promising results from targeted therapies like BRAF and MEK inhibitors offer a glimpse of hope for improving patient outcomes. However, the variability in response rates and the potential for malignant transformation necessitate vigilant follow-up and individualized treatment plans. Additional research is essential to enhance and refine the safe and effective management of these lesions. Future studies should focus on identifying reliable biomarkers for early diagnosis, understanding the mechanisms underlying tumor progression and resistance to therapy, and developing novel therapeutic strategies. Collaborative efforts in clinical trials and multicenter studies will be crucial in gathering robust data to inform evidence-based guidelines. Ultimately, advancing our knowledge and treatment of these rare tumors will improve prognosis and quality of life for affected patients.

## Figures and Tables

**Table 1:** Summary of Grade 3 pleomorphic xanthoastrocytoma involving the ventricular system.

**Case**	**Age/sex**	**Location**	**NF1**	**BRAF mutation**	**Treatment**	**Onset of recurrence**
Fu et al. 2010 [6]	52/M	Right lateral ventricular wall and the third ventricle	N/A	N/A	Subtotal resection, postoperative radiotherapy and chemotherapy	Alive, 6 months after surgery
Roberti et al. 2018 [11]	65/M	Intraventricular centered on the septum pellucidum and extends into the lateral ventricles	N/A	N/A	Gross total resection, followed by palliative Tx	Recurrence after 3 months
Bettencourt et al. 2023 [4]	33/M	Right frontal intraventricular lesion	N/A	Positive BRAF V600E mutation	Surgical resection (not clear if total vs. subtotal), followed by radiation and chemotherapy	Recurrence after 7 months
This case	30/F	Centered in the trigone of the left lateral ventricle with enhancing and nonenhancing components	Yes	Absent BRAF V600E mutation	Subtotal resection × 2 Followed by chemotherapy and radiation therapy	Recurrence 5 weeks postop

## Data Availability

Some data are available upon request due to ethical considerations. Interested researchers can contact the corresponding author at Mohamed.alhantoobi@medportal.ca to request access to the data. Data will be shared in accordance with institutional guidelines and after obtaining necessary permissions.
